# Non-A Non-B Acute Aortic Dissection: Is There Some Confusion in the Radiologist’s Mind?

**DOI:** 10.3390/tomography9060174

**Published:** 2023-12-15

**Authors:** Tullio Valente, Giacomo Sica, Federica Romano, Gaetano Rea, Roberta Lieto, Marisa De Feo, Alessandro Della Corte, Salvatore Guarino, Candida Massimo, Mariano Scaglione, Emanuele Muto, Giorgio Bocchini

**Affiliations:** 1 General Radiology Unit, AORN Ospedali dei Colli, Monaldi Hospital, 80131 Naples, Italy; gsica@sirm.org (G.S.); sag1981@libero.it (S.G.); giorgio.bocchini@gmail.com (G.B.); 2 Department of Translational Medical Sciences, Vanvitelli University, Monaldi Hospital, 80131 Naples, Italy; 3 Department of Medicine, Surgery and Pharmacy, University of Sassary, 07100 Sassari, Italy; mscaglione@uniss.it

**Keywords:** non-A non-B aortic dissection, acute aortic disease, computed tomography angiography, TEVAR, management

## Abstract

Background: The aim of this study is to define and determine the rate of acute non-A–non-B aortic dissections, and to evaluate CT angiography findings and possible complications, as well as to discuss management strategies and currently available therapy. Non-A non-B type of aortic dissection is still a grey area in the radiologist’s mind, such that it is not entirely clear what should be reported and completed in terms of this disease. Methods: A retrospective single-center study including 36 pre-treatment CT angiograms of consecutive patients (mean age: 61 years) between January 2012 and December 2022 with aortic dissection involving the aortic arch with/without the thoracic descending/abdominal aorta (type non-A non-B). Results: According to the dissection anatomy, we identified three modalities of spontaneous acute non-A–non-B anatomical configurations. Configuration 1 (*n* = 25) with descending-entry tear and retrograde arch extension (DTA entry). Configuration 2 (*n* = 4) with Arch entry tear and isolated arch involvement (Arch alone). Configuration 3 (*n* = 7) with Arch entry and anterograde descending (±abdominal) aorta involvement (Arch entry). CT angiogram findings, management, and treatment options are described. Conclusions: Acute non-A non-B dissection represents an infrequent occurrence of aortic arch dissection (with or without involvement of the descending aorta) that does not extend to the ascending aorta. The complete understanding of its natural progression, distinct CT angiography subtypes, optimal management, and treatment strategies remains incomplete. Within our series, patients frequently exhibit a complex clinical course, often necessitating a more assertive approach to treatment compared to type B dissections.

## 1. Introduction

The advancements in noninvasive cross-sectional imaging techniques, such as volumetric CT angiography (CTA), have enabled the swift detection of acute aortic dissection (AD) in the emergency room, making the interpretation of imaging findings by the radiologist an indispensable aspect of diagnosis [1,2,3,4].

The most commonly used classifications for AD management, focused only on the results of surgery/catheter angiography and developed to facilitate triage and improve patients’ treatment results, were introduced over 50 years ago, and rely on the following:

− The extent of dissection or presence of ascending (ATA) or descending thoracic aorta (DTA) flap, without considering the site of primary entry tear (ET) (Stanford classification, 1970) [5];
− The site of primary ET/AD origin (DeBakey classification, 1965) [6].

Dissections involving the aortic arch sparing the ATA are classified as type B in the Stanford system [5]. Over time, the guidelines of the most important European and American scientific societies have stated that if the ATA is acutely dissected (Stanford type A, DeBakey type I and II), the rate of mortality within the first 48 h is greater than 50%, and emergency surgery is necessary. If the dissection involves the DTA-sparing ascending tract (Stanford type B, DeBakey type III), conservative or interventional/endovascular treatment is recommended depending on the morphological/clinical factors and uncomplicated/complicated evolution of the disease [7,8,9,10,11].

Both the Stanford and DeBakey classifications do not clearly address the clinical scenario in which the aortic arch is dissected without involving the ATA, with or without DTA/abdominal dissection, leading to confusion and a major discrepancy in the classification systems typically used by radiologists and surgeons in the clinical practice [12]. Furthermore, these classifications do not account for the more subtle signs today detectable with excellent temporal resolution and isotropic submillimeter spatial resolution cross-sectional imaging [13], and for new endovascular, hybrid or surgical treatment options [14,15,16,17,18]. Though some other management-based AD classifications like PENN (2011) [19], DISSECT (2013) [20] and TEM (Type, Entry and Malperfusion, 2020) [21] tried to clarify AD subtypes, predict outcomes and select optimal treatment, some confusion persists mainly due to guidelines lack for the management of this specific non-A non-B clinical entity.

In 1994, Von Segesser first defined the distal aortic arch dissection as non-A non-B dissection [22].

In 2019, the European Association for Cardio-thoracic Surgery and the European Society for Vascular Surgery released an expert consensus document detailing the management of thoracic arch pathologies. Within this document, an additional category termed “non-A non-B dissection” was introduced by an author, expanding the Stanford classification to include patients whose proximal dissection flap originates in the aortic arch [23]. This statement is also reported in the 2022 ACC/AHA Guideline for the Diagnosis and Management of Aortic Disease [24].

Acute non-A non-B AD type is an aortic arch dissection (primary entry tear or retrograde flap between the brachiocephalic trunk (BCT) and the left subclavian artery (LSA)) not accompanied by the involvement of the ascending aorta, with or without DTA or thoraco-abdominal aorta involvement [23,25,26,27]. In these limited cases, the site of dissection is confined to the aortic arch or begins distally at the descending aorta reaching the aortic arch in a retrograde pathway, sparing the ascending aorta [28,29,30,31].

The objective of this retrospective study is to analyze the CTA incidence, anatomical configurations, entry sites and outcomes of non-A non-B AD according to a consecutive patient series from a tertiary aortic center and to update radiologist’s community about the available treatment options, shedding some diagnostic light on this doubtful topic.

## 2. Materials and Methods

### 2.1. Study Population

This retrospective study was approved by the local Institutional Review Board (AOC-0004415-2021) and conducted according to the principles of the Declaration of Helsinki. In consideration of the retrospective nature of the study, informed consent was waived.

Terms such as “acute aortic dissection”, “aortic arch dissection” and “retrograde type B aortic dissection” were searched in our Radiology Information System (RIS) for all patients with acute AD arrived at our tertiary care hospital between January 2007 and December 2019. Medical histories and surgical results were collected from medical and surgical records. We identified and retrospectively re-evaluated good-quality CTA examinations of the chest, abdomen, and pelvis at initial presentation with aortic arch dissection without ATA involvement, confirmed for all patients by transesophageal echocardiography. Suboptimal CTA studies, iatrogenic and traumatic aortic arch dissection, chronic dissections with acute extension, and aneurysms were excluded.

#### CTA Technique and Image Analysis

The CTA technique has already been described in a previous report [3].

**Figure 1:**
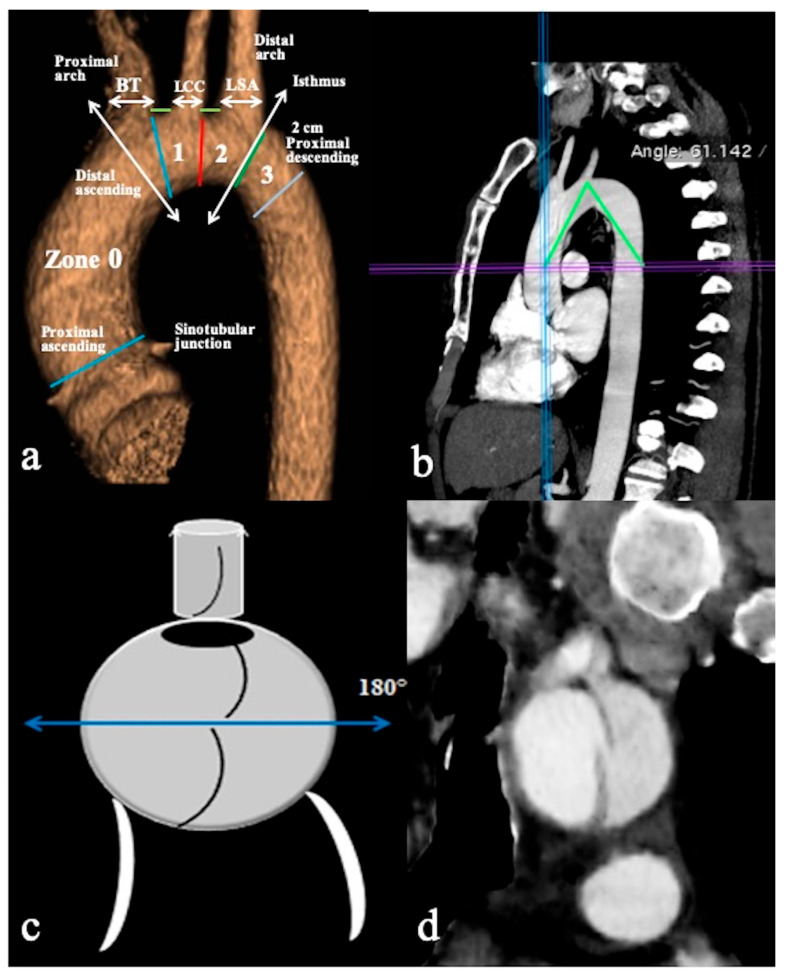
CTA of normal aortic arch provides an overview of aortic landmarks. (**a**) A parasagittal 3D volume-rendering reconstruction of the aortic arch obtained using the high-pitch helical mode (2 × 128-slice CT scanner) without ECG gating and demonstrating arch landmarks. (**b**) The aortic arch angle is defined as the angle formed between a line connecting the highest point of the aortic arch and a mid-luminal point of the ascending and descending aorta at the level of the bifurcation of the pulmonary trunk (horizontal axis) in the parasagittal plane. (**c**) Schematic overview and (**d**) coronal CTA reconstruction image depicts the primary entry tear located at the upper circumference (180°) of the aortic arch’s convexity, while the remaining tears are identified at the arch’s concavity. Specific markers include the Brachiocephalic Trunk (BCT), Left Common Carotid Artery (LCCA), Left Subclavian Artery (LSA), and short green lines indicating the distance between the BCT and LCCA, as well as the distance between the LCCA and LSA.

All CTA scans were performed on a 64-slice (Light Speed VCT; GE Healthcare Waukesha, WI, U.S.A. ) or a dual-source 2 × 128-slice (Somatom Drive; Siemens Medical Solutions, Erlangen, Germany) CT scanner with the anatomic coverage from the distal neck through the pubic symphysis and 0.625–1.25 mm slice thickness. DICOM data sets were retrieved from the local picture archiving and communication systems (PACS), deidentified, and uploaded to a DICOM MD viewer software(OsiriX, Pixmeo, Switzerland) in order to obtain 3D multiplanar/curved reconstructions and qualitative/quantitative information. All examinations were always obtained in a plane perpendicular to the manually corrected local aortic centreline (especially in the parasagittal plane for the aortic arch and the take-off of supra-aortic branches (SAB) evaluation), using MIP (Maximum Intensity Projections) and 3D-VR (Volume Rendering) post-processing techniques (Figure 1).

Dissection’s type including the entry tear site, grade of aortic involvement, and the malperfusion status [21,31,32] were carefully analyzed in all patients at admission according to high-pitch CTA and always, when possible, to ECG-gated CTA. All optimized dose-saving CTA acquisitions were first independently and blindly reviewed by three experienced radiologists with 8–20 years of cardiovascular imaging experience, and then collectively discussed to reach a consensus. Aortic diameters were calculated as the mean of maximum and minimal diameter. The entry tear and eventually re-entry tears were described according to their location (arch concavity, arch convexity) and the grade of aortic arch involvement according to zonal arch anatomy; moreover, the presence of angulation or tortuosity in arch morphology and distances between SAB on arch convexity were also evaluated. Other main findings were aortic flap extension, false lumen patency, lumina pressure competition and diameters, SAB and cerebral and abdominal vessel involvement.

### 2.2. Statistical Analysis

Discrete data are given as counts and percentages. Categorical variables are displayed as frequencies. Denominators solely represent the reported actual cases, and any absent data were not assumed as negative. Continuous variables exhibiting normal distributions are depicted as mean ± standard deviation, while those displaying skewed distributions are shown as median with first and third quartiles.

Data analyses were performed using Microsoft Excel Version 16.11.1 (Microsoft, Redmond, Wash).

## 3. Results

The cumulative pre-treatment spontaneous acute CTA AD caseload was *n* = 386. Stanford A was 198 (51%); Stanford B was 152 (39%); non-A non-B was 36 (9%); 26 (72%) non-A non-B patients were males and the mean age at presentation was 61 years (range: from 51 to 78 years). The proximal ET was located in the aortic arch in 11 patients (30%). The age and sex of non-A non-B patients and the initial admission CTA results are summarized in Table 1.

Pursuant to the institutional dissection protocol, all patients initially received intravenous antihypertensive agents with the aim of reducing systolic blood pressure to <110 mm Hg and achieving a heart rate of <70 beats/min.

Conservative or interventive management and outcome were determined by clinical conditions and dissection of CTA anatomy.

### 3.1. Non-A Non-B Dissection CTA Configurations

**Figure 2:**
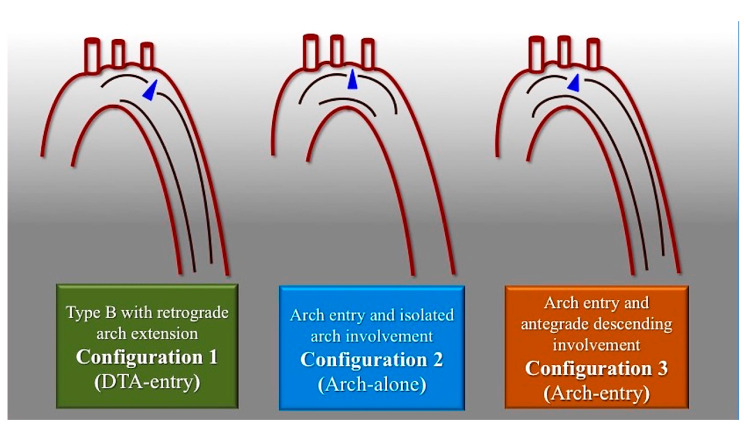
Scheme of non-A-non-B aortic dissection anatomical configurations. Configuration 1 consists of descending entry and retrograde arch extension (DTA entry). In Configuration 2, there is an Arch entry with isolated arch involvement (Arch alone). Configuration 3 consists of an Arch entry and anterograde descending aorta involvement (Arch entry). Blue triangle marks the primary entry tear.

Based on the CTA findings, we classified three distinct anatomical configurations of non-A non-B AD (Figure 2).

**Figure 3:**
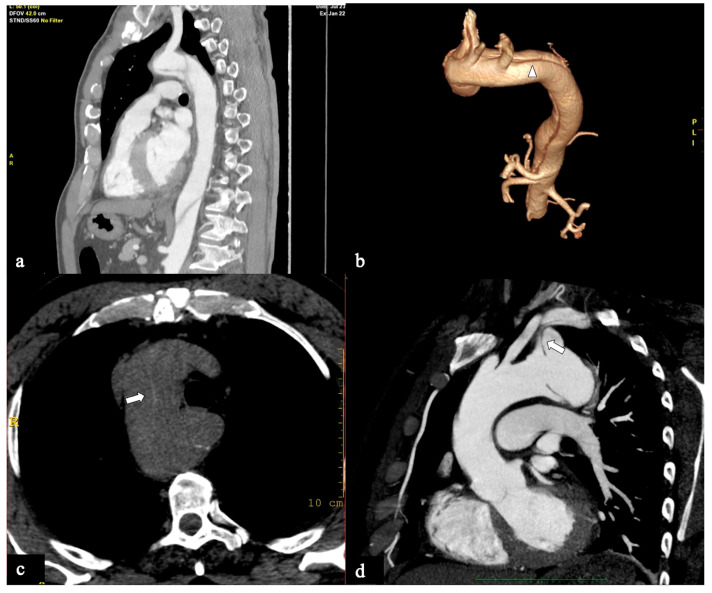
CTA images of non-A–non-B dissection with descending entry and retrograde arch extension or Configuration 1 (DTA entry). (**a**) Sagittal MIP and (**b**) volume-rendering CTA reconstructions in a 74-year-old male showing Configuration 1 dissection with retrograde flap (arrowhead) propagation along the arch curvature. (**c**) In a 65-year-old male patient unenhanced axial CT image shows a hyperdense propagated flap in a right aortic arch (arrow). (**d**) In a 68-year-old female patient curved MIP CTA reconstruction shows left subclavian artery involvement (arrow) in a Configuration 1 dissection.

**Figure 4:**
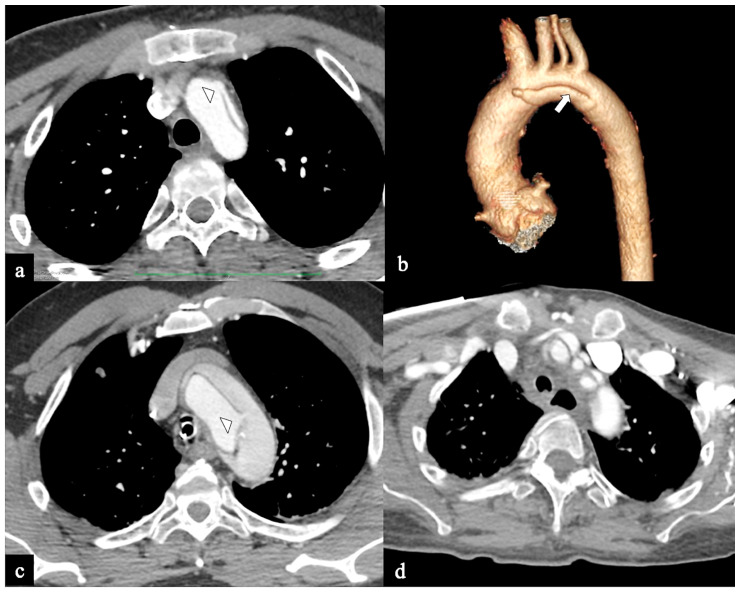
CTA images of non-A non-B dissection with Arch entry and isolated arch involvement or Configuration 2 (Arch alone). A 53-year-old man admitted to the hospital because of severe chest pain. (**a**) Axial CTA image displays the entry tear (indicated by the arrowhead) in the arch convexity. (**b**) A 3D-VR parasagittal reconstruction exhibits a localized arch dissection (arrow) without involvement of the supra-aortic trunks, along with the left vertebral artery originating from the arch (4-vessels arch). The patient received medical treatment. Subsequent CTA examinations conducted at three, six, twelve months, and two years in the follow-up period revealed no progression of the aortic abnormality. (**c**) Axial CTA image shows primary entry tear (arrowhead) in the arch in a 70-year-old man with acute chest pain and stroke. (**d**) In the same patient, a more cranial axial CTA image shows supra-aortic vessel dissection involvement.

**Figure 5:**
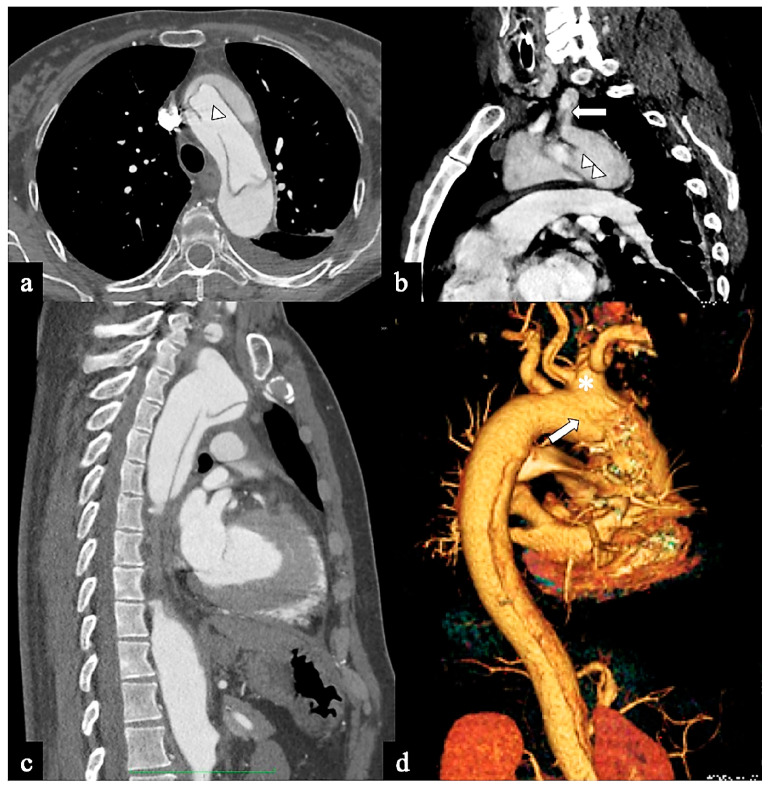
CTA images of non-A non-B dissection with Arch entry and anterograde descending aorta involvement or Configuration 3 (Arch entry). A 68-year-old man was admitted to the emergency room following a sudden onset of intense, sharp back pain accompanied by a syncopal episode. (**a**) Axial CTA image shows a large (>1 cm) primary entry tear in the aortic arch (arrow). (**b**) Sagittal MIP reconstruction shows antegrade flap extension and LSA involvement. A 77-year-old man experiencing severe chest pain and dizziness. (**c**) Oblique parasagittal MIP and (**d**) sagittal volume-rendering reconstructions show bovine arch variant (*), entry tear in arch concavity (arrow) and supra-aortic vessel dissection.

- Configuration 1 (or DTA entry): Among the cohort, 25 out of 36 patients (69.5%) presented with a type B AD or descending-entry type, with the primary entry tear distal to the left subclavian artery (zone 3). The extension of the flap occurred antegradely into the descending aorta, with or without the involvement of the abdominal aorta, and retrogradely into the aorta until the brachiocephalic trunk (BCT) (Figure 3). We describe this configuration as non-A non-B dissection with a retrograde pathway of dissection, as the entry site of the flap is in the descending aorta with retrograde arch involvement. According to Stanford classification, this configuration was present in 14% of type B AD patients. Previous studies have documented DTA entry type to be present between 10% and 25% of patients with acute type B dissection, sometimes as an intramural hematoma [33,34,35]. Patients presenting with DTA entry non-A non-B dissection demonstrated a prevalent origin of BCT and LCCA in 20% and an arch origin of the left vertebral artery in 4%. Among these cases, the distal extent of dissections was observed in various locations: the thoracic aorta in 9 patients, the abdominal aorta in 7 patients, and the pelvic circulation in 9 patients. Involvement of specific arteries was noted, including the left subclavian (*n* = 5), left common carotid (*n* = 1), or innominate (*n* = 1) arteries. Six of these DTA entry patients, who were initially managed with medical therapy, necessitated hybrid or surgical aortic repair within 10 days after the onset of dissection due to persistent pain (3/5), new organ malperfusion (2/5), or rapid aortic growth (1/5). Forty percent of these patients underwent treatment with optimal medical therapy and close imaging surveillance, while 60% received hybrid or open surgical therapy.

- In Configuration 2, the primary ET is situated within the aortic arch, and the dissection flap is confined solely to the arch (Arch alone). the primary ET is located in the aortic arch and the dissection flap is limited to the arch (Arch alone). This very uncommon configuration occurred in 4 (11%) patients (Figure 4) and is a very rare clinical entity. Configuration 2 is similar to the “group B” definition provided half of a century earlier by Dubost and colleagues [36], reported by Pasic in 1999 [37], and again proposed by Urbanski in 2016 [26]. An amount of 2/4 of these Arch alone patients received continued medical management with close imaging surveillance, and one- and two-year follow-up CTA examinations showed no dissection progression; 2/4 patients underwent hybrid or open surgical repair, one due to persisting pain, and the other one for cerebral malperfusion on follow-up CT.

- In Configuration 3 (Arch entry), the primary ET is situated within the aortic arch, and the flap extends into the DTA without affecting the ascending aorta [38]. This Configuration 3 occurred in 7 (19%) patients (Figure 5a,b). This AD configuration was already described as proximal type B dissection according to 2010 AHA guidelines [7] and recently reported as arch B group AD from IRAD [38] and as non-A non-B acute AD with entry tear in the aortic arch [39,40]. In this configuration, the ET was often located in the greater curvature of the arch (4/6), while in one case (1/7) its location was not clearly identified. In total, 4/7 of these patients underwent hybrid and 2/7 surgical therapy.

### 3.2. CTA Findings

According to primary or proximal ET location diagnosis, the CTA sensitivity was 91% for the aortic arch in all comers vs 96% for DTA tear (Configuration 1). In all cases, the primary ET was not clearly visible in 2 (9%), arch variants were present in 9 (25%) (Figure 5c,d) and a shared origin of the BCT and LCCA was seen in 7 (19%), whereas an origin of the left vertebral artery from aortic arch was seen in 3 (8%) because 1 patient showed both “bovine” arch conformation and independent origin of left vertebral artery. A right arch was present in 1 patient. The remaining CTA findings of aortic dissection were similar to those already described and well known [1,2,3,4,32]

**Figure 6:**
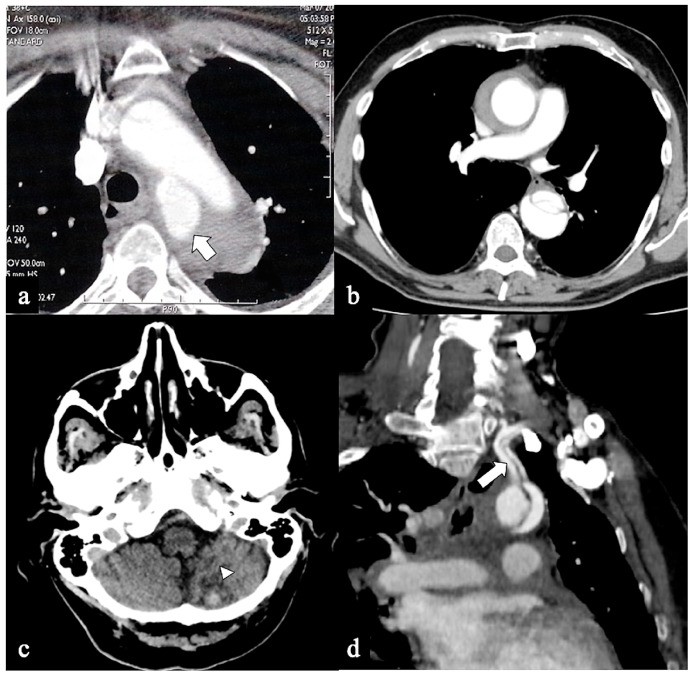
CTA of non-A non-B dissection early complications. A 77-year-old man transferred from another hospital because of acute type B dissection with retrograde flap extension (DTA entry) diagnosis. (**a**) Admission axial CTA image shows an arch ulcer-like projection (arrow) and intramural hematoma. (**b**) In the same patient axial CTA image showing ascending aorta intramural hematoma by retrograde type A aortic dissection. A 69-year-old man with acute chest pain and a recent spontaneous fall on the street. (**c**) Unenhanced head emergency CT axial image shows hemorrhagic stroke in the left PICA territory (arrowhead). (**d**) In the same patient, coronal CTA reconstruction shows LSA dissection in Configuration 1 (DTA entry) non-A non-B dissection (arrow).

About 60% of non-A non-B type AD had a complicated clinical course and required more aggressive or emergency intervention. Medical treatment was globally performed in 13 (36%) patients presenting only with controlled hypertension or gradually disappeared pain. Operative management (endovascular, hybrid or surgical treatment) was performed in 23 (64%) patients presenting with persisting pain or signs of end-organ malperfusion, overt or contained aortic rupture, renal or cardiorespiratory failure. In-hospital AD acute major complications were 2 (5%) represented by the retrograde extension of dissection (retrograde type A dissection) in Configuration 1 patients and arch aneurysm contained rupture in Configuration 3. Malperfusion as defined by clinical, laboratory and imaging evidence (cerebral *n* = 1, gastrointestinal *n* = 1, renal = 2, ilio-femoral *n* = 1) was observed in 5 (14%) patients (3 in Configuration 1; 1 in Configuration 2; 1 in Configuration 3) (Figure 6). A total of 2 (5%) patients presented with clinical and imaging signs of impending rupture or rupture (*n* = 1 in Configuration 1; *n* = 1 in Configuration 3), requiring timely treatment. About half of the patients (52%) underwent some form of TEVAR/hybrid treatment (44% TEVAR with extra-anatomic by-pass, 33% TEVAR with chimney graft and 22% for hybrid arch repair and zone 0 TEVAR). “Hybrid techniques” usually involved sternotomy with arch debranching, thereby creating a proximal landing zone of adequate length, followed by stenting over the arch [11,41]. Major surgery was (*n* =1) en bloc total arch replacement, (*n* = 1) elephant trunk, and (*n* = 2) frozen elephant trunk (FET). In acute non-A non-B patients stroke rate was 11%, the paraplegia rate was 5%; overall in-hospital mortality was 17% (6/36 patients).

## 4. Discussion

Curved morphology of the aortic arch and its branches offspring on the arch convexity, especially the BCT and LSA, appear to constitute natural barriers to the dissection extent, and this may account for the lower incidence of arch involvement in dissections in our series (9%), reported in 4–25% of patients with acute Stanford type B AD in other series [7,25,26,27,28,29,30,31,33,34,35,42].

The variability of these reported data is probably related to discrepant definitions. 

Indeed, there is no consensus on the term “arch extension”, as some authors define it only if the intimal tear originates in the arch while other authors consider only the aortic arch involvement by the flap, independently from the site of the primary tear. 

Moreover, the Ishimaru zones, delimited by brachiocephalic artery origin [43,44] must be applied for the correct definition of arch anatomy.

Given these premises, the goal of the present study is to offer a comprehensive examination of non-A non-B acute AD, analyzing the CTA incidence, anatomical configurations, entry sites and outcomes in a consecutive cohort of patients admitted to our hospital with acute aortic syndrome. We propose three main configurations as a simple anatomical approach that seems related to several outcomes, too.

Similar to type-A and type-B AD, the treatment objectives should prioritize saving patients’ lives, sealing the ET, and facilitating favorable remodeling of the downstream aorta. Currently, consensus has not been reached regarding the optimal treatment strategy for every form of dissection extending into the arch. Such approaches must be individualized based on the morphology of the dissected aorta, discerning between distal lesions (involving LSA) and more proximal ones (involving BCT and LCCA). In the present era, available treatment modalities encompass open surgery (involving replacement of the standard aortic arch or utilization of the frozen elephant trunk (FET) technique), endovascular repair of the thoracic aorta (utilizing TEVAR with chimney stent graft or extrathoracic surgical transposition of supra-aortic branches), or a hybrid procedure (involving debranching of the supra-aortic vessels alongside stent placement). Patients displaying no indications of complications such as aortic rupture, compression of supra-aortic branches (SAB), or end-organ malperfusion, might initially receive conservative management. However, both our experience and that of others suggest potential benefits from early intervention (e.g., TEVAR ± endovascular or surgical debranching) [28,38]. According to the International Registry of Acute Aortic Dissection (IRAD), retrograde arch B type dissection (our Configuration 1/DTA entry) does not affect management strategy or early (in hospital) and late 3 years mortality [33,35]. Conversely, a study by Erbel et al. reported a poorer survival rate in retrograde extension of type III AD [45]. 

Valentine et al. found a higher rate of heart and neurological complications in B-type AD with arch involvement, requiring a prompt intervention, and AD-related deaths [46].

These DTA entry patients were more likely to be involved with arch vessel involvement in our series (28% vs. 10.6% in 2007 IRAD results) [33]. In our patients, primary ET in the arch worsened outcome, and this must be considered as a sign of advanced dissection instability, needing to be more aggressively treated. Unstable patients with non-A non-B AD Configuration 1 are in a gray zone between endovascular and hybrid treatment because the landing zone for a standard endovascular repair (TEVAR) is not a healthy aortic segment and an adequate proximal landing zone. The treatment should be theoretically tailored to the morphology of the dissected aorta with TEVAR for more distal arch lesions and open arch replacement or hybrid arch repair or frozen elephant trunk (FET) for more proximal ones or if there is an Arch entry type. Ideally, zone 1 should remain uninvolved in dissections, and a sufficient distance between the origin of the LCCA and LSA is crucial for effectively sealing the lesion and ensuring the safety of the procedure. Endovascular repair, utilizing a proximal landing zone in zone 1, can be achieved using two Chimney grafts (ch-TEVAR) or with a single chimney graft for the LCCA and a LCCA to LSA bypass or transposition. However, these procedures carry notably higher risks of stroke, retrograde type A aortic dissections, and type Ia endoleak (with an incidence rate ranging from 1.2% to 20%). This endoleak occurs due to incomplete adherence of the aortic stent graft to the chimney stent graft and the aortic wall. Consequently, such interventions should be reserved for patients deemed unsuitable for open surgery or as a last-resort procedure in life-threatening situations when no other viable options are available [47,48,49,50]. Therefore, radiologists should focus on the detection of primary ET localization (proximal/distal) and the curvature involved (convexity/concavity), as well as on SAB flap extension and lumina patency/thrombosis. However, visualization of the primary ET was reported to be difficult, even using excellent temporal resolution multidetector CT scanners [51]. The CTA direct finding of ET is the presence of defect (discontinuity, gap) in the intimal flap shadow, the intimomedial rupture sign [52,53]. Tiny flap defect is sometimes difficult to identify in the axial section and it can be better appreciated with the help of multiplanar reformation. A second useful indirect CTA finding is an abrupt change of false lumen patency (i.e., thrombosed proximally and patent distally). In our series, tears rarely were not clearly displayed (2/36) because of calcifications, and contrast or motion streak artifacts in not ECG-gated CTA. Obviously, the optimization of the CTA scanning protocols and the subsequent different contrast agent concentrations into the two lumina in the arterial phase is a critical factor in obtaining an excellent view of tears and flaps [54]. To date, it remains unclear if the ET location on the arch concavity/convexity may affect non-A non-B AD management and outcome [55,56]. Conversely, it is currently clear that in case of an ET diameter equal to or greater than 10 mm at initial presentation, further false lumen growth is likely [55,56]. The majority of our patients with acute non-A non-B AD presented complications requiring endovascular or surgical treatment. Depending on tear zonal location and if and which SAB are involved, the hybrid treatment of the AA in non-A non-B AD refers to a wide combination of surgical SAB revascularization and TEVAR sealing of the AA. “Hybrid techniques” usually involve sternotomy with arch debranching, thereby creating a proximal landing zone of adequate length, followed by stenting over the AA [47,57,58,59]. If the tear is located in Zone 2, TEVAR should be performed if technically feasible. If the tear is more proximal or Zone 2 is of inadequate length, and/or a concomitant ascending or arch aneurysm is present, then ascending/total arch replacement with hybrid techniques such as FET or arch inclusion technique [16,17,18,60] could represent the optimal therapy. Another option is total endovascular arch repair, which uses fenestrated or branched stent grafts to promote blood flow to the SAB. The FET procedure helps to resolve downstream malperfusion, enables positive aortic remodeling, and provides a stable proximal landing zone for potential secondary TEVAR [61,62,63].

This study shows several weaknesses; first of all, it lacks a statistical analysis that may give greater strength to our results, and, second, other aspects, such as the marked prevalence in men of AD non A non B (72%), deserve future considerations. The agreement between observers in the categorization of the three configurations proposed also appears worthy of evaluation, to reach a tailored radiological report.

## 5. Conclusions

Non-A non-B aortic dissection is an infrequently documented condition, including only a small proportion of all ADs. We have highlighted acute non-A non-B dissection patients’ younger age, associated anatomic arch anomalies, and a substantially higher risk of stroke and aortic rupture when compared to type B dissection. The most suitable approach must be customized for each specific case, relying on precise CT data regarding the dissection configuration, associated complications and the patient’s clinical condition. Thus far, there remains an absence of consensus regarding the optimal management of non-A non-B dissections, leading to persistent controversy on this subject. At present, given the safety advancements in open and endovascular surgeries, there might be consideration for a more proactive approach in the early treatment of non-A non-B dissections.

## Figures and Tables

**Table 1:** Non-A non-B aortic dissection CTA admission results and management.

Variables	All	Configuration 1	Configuration 2	Configuration 3
AD type	*n* = 36	DTA entry = 25 (69%)	Arch alone = 4 (11%)	Arch entry = 7 (19%)
Age	61 ys (51–78)	75 ys	54 ys	69 ys
Sex	M = 26 (72%)	M = 18 (72%)	M = 3 (75%)	M = 5 (71%)
Arch variants	9 (25%)	6 (24%)	1 (25%)	2 (28%)
Arch type - I - II - III	9 (25%) 20 (55%) 7 (19%)	5 16 4	2 1 1	2 3 2
Primary ET diagnosis	34 (94%)	24 (96%)	4 (100%)	6 (86%)
Primary ET location	16 (66%) concavity 18 (75%) convexity	12 (50%) 12 (50%)	2 (50%) 2 (50%)	2 (33%) 4 (67%)
Aortic diameter (mean value, mm) - Ascending - Arch - DTA	44 (±5) 34 (±4) 33 (±3)	43 (±4) 33 (±3) 32 (±3)	43 (±2) 34 (±3) 31 (±3)	45 (±3) 35 (±3) 33 (±2)
SAB dissection - BCT - LCCA - LSA	12 (33%) 2 3 7	7 (28%) 1 1 5	1 (25%) 0 0 1	4 (57%) 1 2 1
Aorta rupture	2 (5%)	1 (4%)	0/4 (0%)	1 (14%)
Malperfusion	5 (14%)	3 (12%)	1 (25%)	1 (14%)
Treatment - Medical - Endovascular - Hybrid - Surgery	13 (36%) 6 (17%) 13 (36%) 4 (11%)	10 (40%) 6 (24%) 8 (32%) 1 (4%)	2 (50%) 0 (0%) 1 (25%) 1 (25%)	1 (17%) 0 (0%) 4 (57%) 2 (26%)
In-hospital acute major complications	2 (5%)	1 (4%)	0 (25%)	1 (14%)
30 days mortality	6 (17%)	3 (12%)	1 (25%)	0 (0%)

Legend: AD (aortic dissection), ET (entry tear), DTA (descending thoracic aorta), SAB (supra-aortic branches), BCT (brachiocephalic trunk), LCCA (left common carotid artery), LSA (left subclavian artery).

## Data Availability

The authors confirm that the data supporting the findings of this study are available within the article.
